# Epiglottitis Strikes Twice: A Case of Adult Recurrent Epiglottitis

**DOI:** 10.7759/cureus.56940

**Published:** 2024-03-26

**Authors:** Brooke Escoe, Brody M Fogleman, Robert Sherertz

**Affiliations:** 1 Department of Internal Medicine, Grand Strand Regional Medical Center, Myrtle Beach, USA; 2 Department of Internal Medicine, Edward Via College of Osteopathic Medicine - Carolinas, Spartanburg, USA

**Keywords:** epiglottitis risk factors, hypogammaglobulinemia, cd4+ lymphopenia, adult epiglottitis, recurrent epiglottitis

## Abstract

Epiglottitis is an uncommon condition in adults, and recurrent episodes are rare. We report a 58-year-old male who had a second episode of epiglottitis nine years after his first. Our patient’s immunologic profile obtained during his hospitalization revealed a significantly low absolute cluster of differentiation 4+ (CD4+) T lymphocyte count of 77 cells/mcL and a low immunoglobulin G (IgG) level of 635 mg/dL. Our patient was successfully managed with broad-spectrum antibiotics and corticosteroids. Given the known ability of short-term corticosteroids and acute inflammation’s effect on lymphocyte populations, the significance of these laboratory values remains unclear due to our patient’s unwillingness to undergo further diagnostic testing following discharge from our facility. We have considered multiple underlying etiologies for our patient’s predisposition to developing this rare, recurrent, infectious manifestation; however, the exact cause is yet to be fully elucidated.

## Introduction

Epiglottitis most commonly occurs when a respiratory infection causes inflammation and swelling of the epiglottis. Historically, the most commonly isolated organism causing epiglottitis in pediatric patients was *Haemophilus influenzae *serotype b (Hib), until the development of an effective vaccine [1]. The Hib conjugate vaccine has been used in infants since the 1980s and has been associated with as much as a 200-fold reduction in pediatric epiglottitis cases (4.9 cases/100,000 to 0.02 cases/100,000 in Denmark) [1,2]. During that same time frame, the incidence of epiglottitis in adults has remained relatively constant (2-4 cases/100,000) [2]. In one large series of 331 adults with epiglottitis, only 4% of adults had a recurrence [3], suggesting that the risk of recurrent epiglottitis in adults is approximately 1 case/1,000,000.

The most common bacteria causing adult epiglottitis are *Streptococcus pneumoniae*, other streptococcal species, *Staphylococcus aureus*, and non-Hib *Haemophilus influenzae* serotypes [1]. The clinical presentation of epiglottitis in adults is different and includes severe sore throat and pain with swallowing, as compared to the stridor and drooling frequently seen in pediatric cases [1]. The risk factors for recurrent epiglottitis in adults are not well established but may include alcohol consumption and diabetes [3]. Very little is known about the possible role of immunodeficiency in recurrent epiglottitis.

Although opportunistic infections are well known to be associated with immunosuppressed patients, the relationship between immunosuppression and recurrent episodes of epiglottitis is not widely reported. In particular, the literature regarding recurrent episodes of epiglottitis in patients with a severe cluster of differentiation 4+ (CD4+) T cell lymphopenia, with or without human immunodeficiency virus (HIV) infection, is limited [4-7]. One case report does describe two patients, both of whom had CD4+ lymphopenia and were found to be HIV-positive, who presented with recurrent episodes of epiglottitis due to ulcerative epiglottic lesions [6]. The exact relationship between the severity of CD4+ lymphopenia and recurrent episodes of epiglottitis, whether HIV-related or not, is unknown.

This case involves a 58-year-old male who previously had one episode of epiglottitis nine years prior to the recurrent episode we discuss herein.

## Case presentation

The patient was a 58-year-old male who presented to the emergency department (ED) with progressively worsening fever, deep throat pain, and malaise for three days. Over this same time frame, he reported odynophagia, voice changes, and facial swelling. His past medical history was significant for hypertension, type 2 diabetes mellitus, gastroesophageal reflux disease, dyslipidemia, and one episode of bacterial epiglottitis nine years prior. He denied any other significant illnesses or hospitalizations in the nine-year time span between episodes. Surgical history was significant for tonsillectomy. Physical examination findings were significant for tenderness to palpation on the anterior neck. Vital signs at the time of admission revealed a fever of 39.3°C (102.8°F), tachycardia of 134/minute, and 99% oxygen saturation on room air, clearly meeting sepsis criteria based on vital signs alone. Laboratory results showed mild leukocytosis of 12,800/mm^3^. Rapid testing was negative for influenza A/B, SARS-CoV-2, group A *Streptococcus*, and methicillin-resistant *Staphylococcus aureus* (MRSA). His at-home medications included sitagliptin/metformin, insulin glargine, empagliflozin, atorvastatin, lisinopril, cyanocobalamin, ubidecarenone, cholecalciferol, biotin, famotidine, lactobacillus combination probiotic, and a multivitamin. All at-home medications were discontinued upon admission, and he was started on an insulin sliding scale regimen.

**Figure 1:**
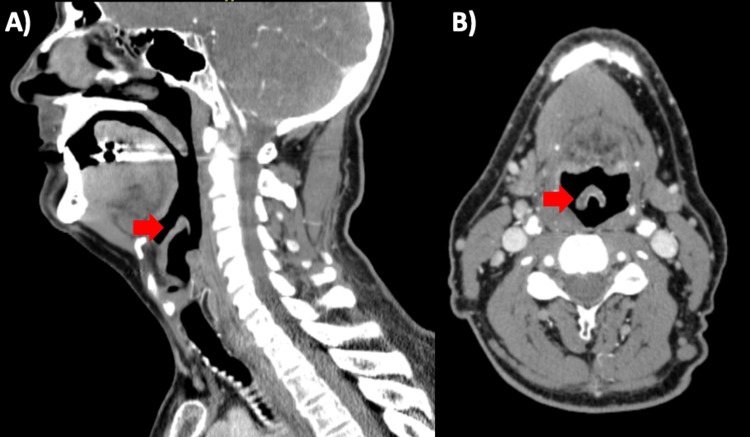
CT imaging of the head and neck depicts asymmetric swelling of the epiglottis (red arrows) in the sagittal (A) and axial (B) planes, most prominent on the right. CT: computed tomography

A computed tomography (CT) scan of the neck with contrast was obtained in the ED and demonstrated asymmetric thickening of the right aryepiglottic fold, as depicted in Figure 1. He was initially given intravenous (IV) clindamycin 900 mg, IV dexamethasone 10 mg, and IV vancomycin 1,750 mg. Fluid resuscitation with Lactated Ringer’s was also initiated. He was then admitted to the medical critical care unit for close airway monitoring, and otolaryngology was consulted.

**Figure 2:**
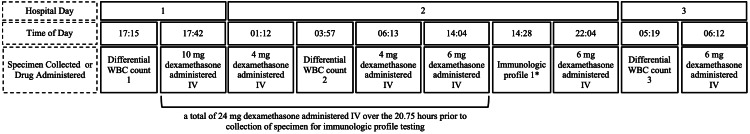
Timeline of events including specimen collection and IV dexamethasone administration. *Laboratory results following patient discharge from the facility WBC, white blood cell; IV, intravenous

On his second hospital day, he was given IV ceftriaxone 2,000 mg, IV ampicillin-sulbactam 3 g, IV vancomycin 1,000 mg, oral (PO) valacyclovir 1,000 mg, and albuterol-ipratropium as needed. The patient was given IV dexamethasone to reduce his throat swelling and prevent progression to respiratory distress, to which he responded well. Figure 2 illustrates the timeline of IV dexamethasone administration and specimen collection throughout our patient’s admission. Table 1 shows the results of white blood cell (WBC) counts with differentials along with the immunologic profile obtained.

**Table 1:** WBC count with differential throughout the patient’s hospitalization revealed elevated neutrophils with low lymphocyte counts. Immunologic testing from day 2 revealed a significantly low absolute CD4+ T lymphocyte count and low IgG level. Bolded values indicate that the value is outside of the normal reference range for the respective parameter. *Laboratory results following patient discharge from the facility WBC(s), white blood cell(s); CD4+, cluster of differentiation 4+; #, absolute cell count; %, relative proportion of specific cell type; IgG, immunoglobulin G; IgA, immunoglobulin A; IgM, immunoglobulin M; N/A, not available

Laboratory parameter	Day 1	Day 2	Day 3	Units	Normal range
WBCs					
#	12.8	11.7	10.9	K/mm^3^	3.7-10.1
Neutrophils					
#	10.9	11.0	9.7	K/mm^3^	1.4-6.5
%	85	93.8	88.6	%	40.1-81.3
Lymphocytes					
#	0.7	0.5	0.8	K/mm^3^	1.2-3.4
%	5.9	3.9	7.2	%	17.0-47.0
Monocytes					
#	1.1	0.2	0.5	K/mm^3^	0.1-0.6
%	8.3	3.9	4.1	%	5.5-11.9
Eosinophils					
#	0.0	0.0	0.0	K/mm^3^	0.0-0.7
%	0.0	0.0	0.0	%	0.0-5.5
Basophils					
#	0.1	0.0	0.0	K/mm^3^	0.0-0.2
%	0.7	0.2	0.1	%	0.0-1.1
CD4+ T lymphocytes					
#	N/A	77*	N/A	cells/mcL	544-1,212
%	N/A	28.9*	N/A	cells/mcL	40.4-57.4
Immunoglobulins					
IgG	N/A	635*	N/A	mg/dL	700-1,600
IgA	N/A	175.8*	N/A	mg/dL	70-400
IgM	N/A	61*	N/A	mg/dL	40-230

His throat pain was well controlled with acetaminophen throughout his hospital stay. Otolaryngology confirmed epiglottitis via flexible laryngoscopy. On day 2, he was doing much better and deemed suitable for transfer out of critical care to the general medicine floor for further management. Blood cultures remained negative. The patient was additionally found to have a low absolute CD4+ T lymphocyte count (77 cells/mcL) and low immunoglobulin G (IgG) (635 mg/dL; normal, 700-1,600 mg/dL), with immunoglobulin A (IgA) and immunoglobulin M (IgM) levels within normal limits (see Table 1).

By the third day of his hospitalization, his fever had resolved, and he had a significant reduction in his sore throat and pain. He was given one final dose of 6 mg dexamethasone and 2,000 mg ceftriaxone and discharged for outpatient follow-up with an otolaryngologist. At discharge, he was prescribed a short course of the following medications: loratadine, nystatin, valacyclovir, dexamethasone, and cefdinir. He was also provided with prescriptions for pneumococcal and Hib vaccines. At follow-up, the patient’s HIV antigen/antibody test was negative.

During the patient’s first episode of epiglottitis, he presented similarly with progressively worsening sore throat and fevers and was able to maintain his airway. Physical examination at that time was remarkable for an erythematous epiglottis with small white spots, which were believed to be *Candida*. The patient was treated with IV antibiotics and IV steroids at that time with uneventful recovery. Given the uncertainty regarding the cause of his first manifestation of epiglottitis, the patient underwent HIV antigen/antibody testing throughout the nine years following the first episode, at least once yearly, each of which returned negative.

## Discussion

The case presented here of recurrent epiglottitis in a 58-year-old male highlights the rarity and complexity of recurrent epiglottitis in adults and expands our understanding of possible risk factors associated with this rare occurrence. The patient’s history of two episodes of epiglottitis raises important questions regarding our current understanding of the role of humoral and cell-mediated immunity in preventing it.

In his second episode of epiglottitis, our patient was treated with high-dose dexamethasone and combination antibiotics to which he responded well over the course of a three-day hospitalization. The administration of 24 mg of IV dexamethasone over the course of approximately 20 hours prior to the specimen collection for CD4+ lymphocyte quantification could have contributed to the low count that resulted. However, there are at least two studies that show that by 24 hours after the administration of IV hydrocortisone at doses of either 50 mg or 250 mg or IV methylprednisolone at a dose of 1 mg/kg, CD4+ lymphocyte counts are either slightly elevated or have returned to normal [8,9]. This, in combination with our routine daily laboratory testing showing no major changes in the absolute WBC counts or the percent, which were lymphocytes in the differential, makes it unlikely that the steroid therapy could account for the extremely low CD4+ lymphocyte count. It was thus much more likely to represent a number close to his baseline level. Another possible contributor to his low CD4+ lymphocyte count was that he met sepsis criteria upon arrival at the hospital. Sepsis has been associated with CD4+ lymphopenia and is also a marker of increased mortality [10,11]. However, in one recent study looking at CD4+ lymphopenia as a marker of sepsis mortality, even those who died and had the lowest CD4+ lymphocyte counts still had median CD4+ lymphocyte counts more than twice as high as our patient [11].

While other factors may have contributed to our patient’s CD4+ lymphocyte count of 77 cells/mcL, it seems likely that he may have had a baseline reason for CD4+ lymphopenia. The most common cause for baseline CD4+ lymphopenia is HIV infection with acquired immunodeficiency syndrome (AIDS). The patient and his spouse had multiple, at least yearly, HIV antibody tests during the nine-year period between his two episodes of epiglottitis, in which all were negative. It is possible that the patient could have HIV infection and be an elite controller, but the patient was unwilling to have an HIV quantitative ribonucleic acid (RNA) viral load test performed. An elite controller is an individual who maintains a low HIV viral load despite not being on antiretroviral therapy, with the majority of those affected having a normal CD4+ T lymphocyte count [12-14]. However, there is a subset of elite controllers who have CD4+ T cell lymphopenia resulting from failed compensatory mechanisms [15]. Some of these patients additionally have negative HIV antibody tests. However, given the repeatedly negative HIV tests over the course of multiple years, it seems less likely that our patient falls into this category. Alternatively, the patient could have other causes of CD4+ T cell lymphocytopenia with concomitant hypogammaglobulinemia. However, our patient’s baseline CD4+ T lymphocyte count is not known in the setting devoid of corticosteroids or infection. Given this unknown, we have considered other possible etiologies that could have resulted in an additive effect in decreasing our patient’s CD4+ T lymphocyte count in the presence of corticosteroid administration.

We also considered the possibility of iatrogenic immunosuppression that could have increased the propensity of our patient to develop this infection; however, none of his at-home medications appear to have any known relationship with immunosuppression. Therefore, in this case, there does not appear to be a good explanation of why our patient developed these recurrent episodes of epiglottitis over such a long time frame without manifestations between. Although it is possible that our patient’s manifestations could have occurred randomly without an underlying immunosuppressive disorder, it is highly improbable given the rarity of this manifestation in the adult population.

Other possible causes of our patient’s CD4+ T cell lymphopenia include a spectrum of underlying conditions that warrant consideration. Chronic viral infections other than HIV, such as human T cell lymphotropic virus (HTLV), may contribute to sustained immune dysfunction and could possibly lead to recurrent or unusual infection risk [16]. Since we did not test for HTLV, we cannot definitively rule it out as a potential cause. Various autoimmune disorders, such as rheumatoid arthritis or systemic lupus erythematosus, could be implicated in CD4+ T cell lymphopenia [16]. However, given that our patient has had no clinical manifestations of an underlying autoimmune disorder throughout the past nine years, this is an unlikely explanation. Additionally, hematologic malignancies should also be considered as a possible etiology [16], although we have no information to suggest this as a likely cause.

Another possible, yet uncertain, consideration for our patient is idiopathic CD4+ lymphocytopenia (ICL), a rare condition characterized by a low CD4+ T lymphocyte count without any identifiable cause [17]. Patients with ICL typically come to clinical attention when presenting with opportunistic or uncommon infections [17]. While epiglottitis itself is not a rare entity, manifestations of such in the adult population are rare. Provided that our patient has now had two episodes of epiglottitis without an identifiable cause, it is necessary to consider a broad differential diagnosis in order to identify the underlying causes that have predisposed our patient to this infection type. In ICL, diagnostic testing commonly reveals the absence of HIV infection and a low CD4+ T lymphocyte count [17]. In this case, our patient does not meet the diagnostic criteria for ICL, as we are unable to completely rule out the potential presence of a subclinical HIV infection, which could be further clarified with HIV RNA viral load testing. Additionally, we would need to clarify whether or not our patient truly has a low absolute CD4+ T lymphocyte count or if the severe decline was solely secondary to corticosteroid administration during his hospitalization. Unfortunately, we are unable to pursue further diagnostic testing on our patient to further refine the underlying cause of his recurrent episodes due to his unwillingness.

The production of antibodies by B cells is facilitated through the interaction with antigen-specific CD4+ T cells, in addition to costimulatory ligands and cytokines, to support B cell proliferation and differentiation in response to recognized protein antigens [18]. This process results in antibodies of the IgG class, among others, resulting in long-term immunity against previously encountered antigens [18]. While a malfunction in the CD4+ T cell-dependent B cell IgG production mechanism appears to be a possible explanation in our patient, the exact cause cannot be refined in the setting of corticosteroid administration along with the lacking information regarding the patient’s true baseline immunologic status. We were unable to obtain follow-up CD4+ T lymphocyte studies or IgG subclass studies to further evaluate for possible underlying immunologic dysfunction. This case is complicated by the fact that corticosteroid administration could have caused the low CD4+ T lymphocyte count and that our lack of ability to perform follow-up testing on our patient restricts us from determining if a true underlying disorder exists.

In a previously published case series including four patients with recurrent epiglottitis, one of the patients was found to have hypogammaglobulinemia without CD4+ T cell lymphopenia [5]. The patient described in that report also had recurrent infections, including severe sinusitis and pneumonia [5], which was not a clinical feature of the patient we present here. If our patient indeed does have a low absolute CD4+ T lymphocyte count and low serum IgG levels in the absence of corticosteroid administration, it could further expand upon observations by Gagnon et al., emphasizing that deficits in humoral immunity may provide an explanation for recurrent epiglottitis in adults [5]. However, our understanding of the exact interplay and the relationships between our patient’s CD4+ T cell lymphopenia, hypogammaglobulinemia, corticosteroid administration, and recurrent epiglottitis discussed herein was limited by the patient’s unwillingness to undergo further testing.

If the patient was willing, we would recommend the following additional evaluations and interventions: 1) HIV viral load, 2) baseline antibody levels for Hib and pneumococci followed by vaccination with the Hib conjugate and pneumococcal conjugate vaccines with follow-up antibody testing, 3) monitoring of CD4+ T lymphocyte counts every six months, 4) quantitative IgG levels every six months, and 5) consistent lifelong follow-up with an infectious disease physician.

## Conclusions

We report a single case of recurrent epiglottitis in an adult male that raises interesting questions regarding the predisposing factors that may have increased his susceptibility to this type of infection. Our patient was found to have severe CD4+ T cell lymphopenia and mild hypogammaglobulinemia during his hospitalization; however, the cause of this derangement is unclear in the setting of corticosteroid administration. Follow-up testing could further clarify whether or not our patient has a true underlying immunodeficiency disorder, yet our patient was unwilling to pursue further diagnostic workup. Adult-onset recurrent epiglottitis is a rare entity and sparsely reported in the literature. It is therefore appropriate in this case to presume that our patient has some dysfunction of his immune system that has increased his risk of recurrent infections of this type. However, at this time, the laboratory results and underlying cause demonstrate an unclear picture and necessitate that a broadened differential diagnosis persists.
